# Dog-assisted therapy on Hong Kong children with autism spectrum disorder: an exploratory randomized controlled trial

**DOI:** 10.1007/s00431-025-06720-6

**Published:** 2026-01-09

**Authors:** Wilfred H. S. Wong, Chen Chen, Amy Tso, Hung Kwan So, Justin P. Y. Wong, Helen Tinsley, Charis H. Y. Chung, Ronda K. W. Luk, Patrick Ip

**Affiliations:** Department of Paediatrics and Adolescent Medicine, School of Clinical Medicine, Li Ka Shing Faculty of Medicine, University of Hong Kong, Room 115, 1/F, New Clinical Building, Queen Mary Hospital, 102 Pokfulam Road, Hong Kong SAR, China

**Keywords:** Dog-assisted therapy (DAT), Autism spectrum disorder (ASD), Quality of life, Psychosocial difficulties, Children

## Abstract

Dog-assisted therapy (DAT) has become a promising complementary approach, providing social and emotional support for children with autism spectrum disorder (ASD). Research on the effects of DAT on multiple functions in children with ASD is limited, and the results are inconsistent. Our study aimed to evaluate the effect of DAT with an RCT study on both psychosocial problems and overall well-being in children with ASD in Hong Kong. An exploratory randomized controlled trial was conducted from February 2023 to November 2024, involving 64 children with ASD aged 6 to 15 years. Participants were randomly assigned (1:1 ratio) to DAT group and control group. The DAT group participated in a structured 8-session DAT training program while the control group received a conventional education curriculum. Before and after the intervention, the Paediatric Quality of Life Inventory scale and Strengths and Difficulties Questionnaire were used to evaluate children’s quality of life and psychosocial problems, respectively. The paired *t*-test and independent *t*-test/Mann–Whitney *U* test were employed to analyze pre-post differences and group differences. In the DAT group, the mean total score of quality of life was significantly improved (pre vs. post: 58.32 vs. 63.71, *P* = 0.007) and the mean score of total difficulties was significantly reduced (pre vs. post: 15.63 vs. 13.16, *P* = 0.003). The decreased scores of several subscales of SDQ, such as externalizing behavior, conduct problems, and hyperactivity were also observed (all *P* < 0.05). The control group with conventional curriculum training also presented with a lowered mean total difficulties score (pre vs. post: 16.47 vs. 15.03, *P* = 0.035). There were no statistically significant change differences between the two groups (*P* > 0.05).

*Conclusion*: The preliminary finding shows DAT has a comparable effect to the school’s educational curriculum in improving the psychosocial health and quality of life of children with ASD. DAT could potentially serve as a beneficial supplemental therapy for children with ASD who receive conventional curriculum training.

*Trial registration*: The trial was registered on https://www.clinicaltrials.gov (NCT06609122) on 25 Sep 2024.

**Table:** 

**What is Known:**
• *Animal-assisted intervention (AAI) has emerged as a promising adjunct, offering social and emotional support for children with ASD*. • *Evidence on the impact of dog-assisted therapy (DAT) on important outcomes (e.g., emotional and behavioral problems, quality of life) were not fully explored*.
**What is New:**
• *DAT demonstrates a similar impact to the school's educational curriculum in enhancing the psychosocial well-being and quality of life of children with ASD*. • *DAT may serve as a valuable complementary therapy for children with ASD alongside conventional curriculum-based training*.

**Supplementary Information:**

The online version contains supplementary material available at 10.1007/s00431-025-06720-6.

## Introduction

Autism spectrum disorder (ASD) is a common neurological and developmental disorder, diagnosed in early childhood. It is characterized by deficits in social communication and interaction, along with repetitive behaviors [1]. It is difficult for children with ASD to establish and maintain relationships, develop social-emotional reciprocity, and elaborate communicative behaviors such as eye contact and body language [2]. Some children with ASD have severe behavioral problems, such as stereotyped or repetitive motor movements, and extreme distress at small changes [2]. These differences and issues affect their relationships, academic performance, and employment prospects [3]. Current interventions for ASD involve educational approaches, developmental therapies, and behavioral interventions [4], but most are expensive and time-consuming [5].

Animal-assisted intervention (AAI) has emerged as a promising adjunct, offering social and emotional support for children with ASD [6, 7]. Horses have been the most utilized species, while there has been a scarcity of research involving dogs and their effectiveness with quantitative studies or well-designed randomized controlled trials (RCTs) [7]. Most studies to date have been qualitative reports providing positive parents’ and therapists’ feedback on the dog-interaction activities for ASD children, noting a decrease in self-aggression and repetitive stereotyped movements, and improvements in communication and creativity [8, 9]. An increasing amount of evidence from qualitative and observational research indicates that dog-assisted therapy (DAT) may improve social functioning in children with ASD, particularly by promoting social approach behaviors and skills and decreasing social withdrawal [10–12]. Methodologically, a pilot study from Spain [10] reported an uncontrolled pre-post pilot study employing observation-based assessments (ACIS and Animal-assisted Therapy Flow Sheet) within a single group of 19 young children (mean age ~ 4 years). The intervention consisted of a median of nine individual, weekly sessions (~ 20 min each), led by an occupational therapist and following a semi-standardized protocol. While the study reported significant improvements in communication and social interaction skills with large effect sizes, the authors highlighted the absence of a control group as a major limitation, making it difficult to attribute the effects solely to the intervention amidst other concurrent therapies. These non-randomized studies, while helpful for forming hypotheses, are prone to bias and lack rigorous controls. The field is progressing toward more rigorous research through RCTs, but current RCTs have certain limitations. For example, one focused solely on social communication outcomes [13], and another involved a very small sample of young children (*n* = 22, aged 4–6) [14], which restricts the broader applicability of their findings.

Besides social interaction and communication [10, 12, 15, 16], other important outcomes (e.g., emotional and behavioral problems, quality of life) were not fully explored. A recent review also emphasized the importance of understanding treatment outcomes and prioritizing the well-being of human subjects [7]. Becker et al. documented emotional symptoms, showing decreased feelings of isolation and overall depressive symptoms, however, the effect of change over time was not significant [17]. Another study showed AAI increased positive gestures and facial expressions in children and improved peer interaction, but the intervention was not well described [8]. Protopopova, A. et al. conducted a case study that observed a reduction in salivary cortisol levels during Dog-Assisted Therapy (DAT) sessions [18]. However, since this was a non-controlled case study, these results should be interpreted with caution. Without a control group or baseline measurements, it is challenging to confidently attribute the physiological changes solely to the DAT, as they could also be influenced by other factors such as the novelty of the session or the overall therapeutic setting [18]. Overall, the exploration of DAT on various functions of children with ASD is limited and the findings remain diverse due to the non-standardized intervention designs and diverse settings.

Assessing the effectiveness of DAT in existing research is further complicated by certain methodological limitations. Primarily, many studies depend on assessments from a single source, such as parent reports, which introduces the risk of reporter bias and fails to fully capture the complex outcomes of therapy [3, 9, 11]. For example, parents and caregivers often note qualitative improvements in areas like bonding, communication, and emotional security [3, 9, 11], while clinicians and therapists may simultaneously observe enhancements in core social-communication skills, such as eye contact and gesturing, within clinical settings [10, 19]. This discrepancy underscores the importance of using multiple informants to obtain a more comprehensive understanding. Additionally, the ecological validity of these findings remains uncertain—specifically, whether the skills learned during DAT sessions transfer to and are maintained in everyday settings like school or community environments outside the therapy context. Importantly, integrating perspectives from various settings—such as clinics, schools, and homes—is rarely done. An exception is the study by [7], which included ratings from therapists, teachers, and the children themselves, highlighting the value of a multi-assessment approach.

To address these research gaps, this study employs a well-structured RCT with a relatively sufficient sample size to examine the effects of DAT on Chinese children with ASD in Hong Kong. Specifically, we aim to address the following research questions: (1) Does DAT significantly enhance psychosocial functioning and quality of life in children with ASD? (2) Are any observed treatment effects maintained across different settings (home and school) as reported by multiple assessments (parents and teachers)? (3) Is DAT better than traditional courses training for improving the mental health of children with ASD. Based on previous literature, we hypothesize that (1) DAT will lead to enhanced psychosocial functioning and quality of life; (2) treatment effects will be consistent across home and school settings as reported by both parents and teachers; and (3) DAT may have better effectiveness in improving children’s mental health.

## Methods

### Study design and population

**Fig. 1:**
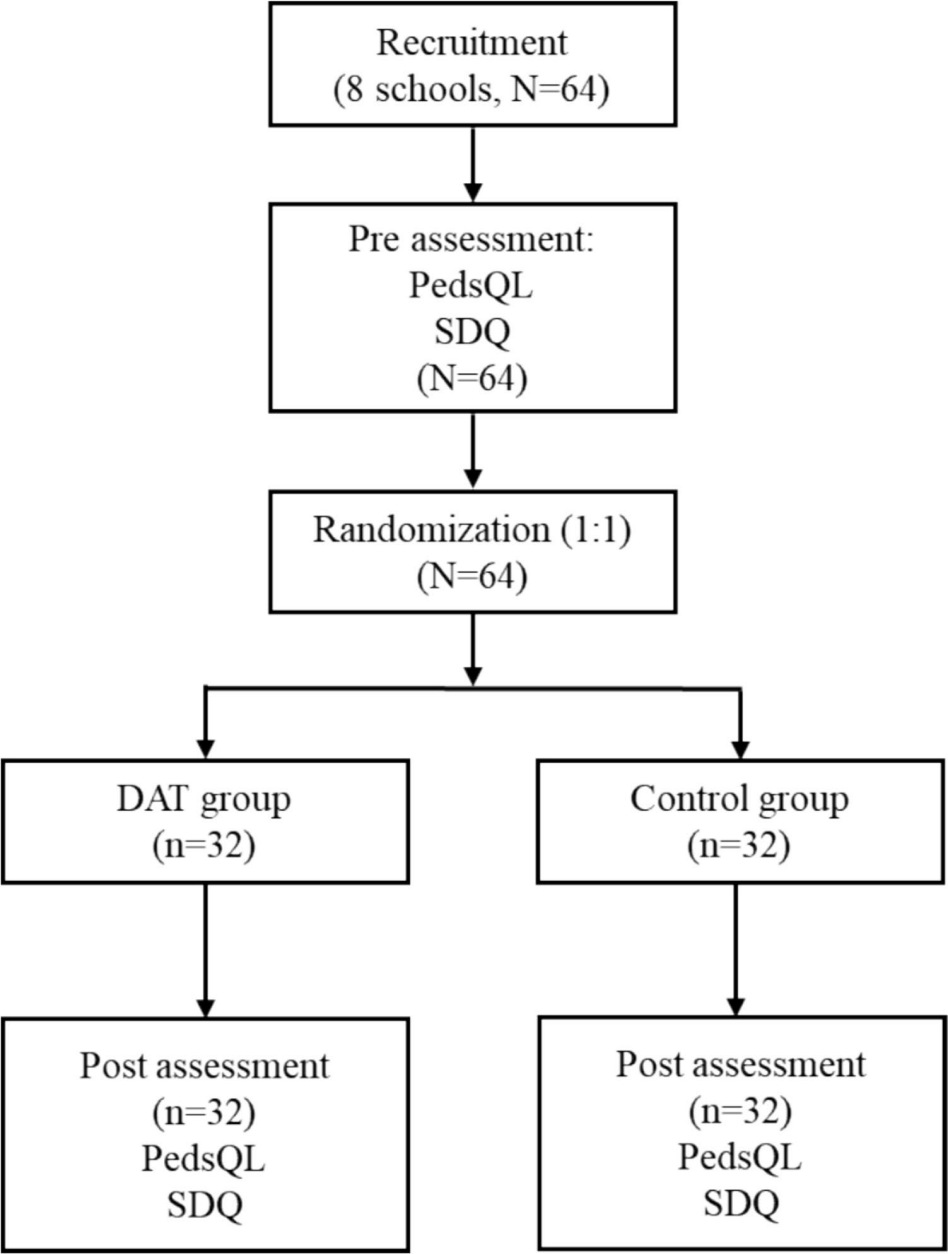
Diagram of the study design. PedsQL: Pediatric Quality of Life Inventory; SDQ: Strengths and Difficulties Questionnaire; DAT: Dog-Assisted Therapy

This was a randomized controlled trial to compare the impact of DAT and conventional curriculum training on children with ASD in Hong Kong. As shown in Fig. 1, from February 2023 to November 2024, 64 children aged 6 to 15 years were recruited from 8 Hong Kong special educational needs (SEN) schools for ASD (primary diagnosis) children with mild to moderate intellectual disabilities (The sample size for this randomized controlled trial was determined primarily by feasibility considerations, acknowledging the recruitment challenges for this specific population in Hong Kong. A power calculation provided a supporting rationale. Based on our pilot study using the Family Functioning Summary Score as a reference (mean difference = 2.6, standard deviation = 3.68), a large effect size (Cohen’s *d* ≈ 0.71) was observed. For 80% power at a 5% level of significance (two-tailed), a total sample of 34 participants (17 per group) would be required to detect an effect of this magnitude using an independent-samples *t*-test. To account for potential attrition, we aimed to recruit a total sample of 64 participants). Before intervention, 64 participants were assessed by questionnaires (PedsQL and SDQ) and were randomized into the DAT and control groups. After intervention, no participants withdrew from the project; all participants joined the post-assessment by the same two questionnaires (Fig. 1). The SEN schools were selected through the Committee on Home School Co-operation in Hong Kong. The block randomization was carried out with blocks of four. Within each block, participants were randomly assigned to the DAT group or the control group with a 1:1 ratio. The random sequence for block randomized allocation was computer-generated. The randomization was performed by a team member who was not involved in the recruitment or assessment of participants, ensuring blinding of group allocation. The therapy dogs were introduced to the SEN school environment and had interactions with the children for 1 week prior to the intervention. This allowed the dogs to acclimate to the school setting and the presence of the children, ensuring a smoother transition into the intervention phase. Inclusion criteria for participants included (1) either gender aged 6 to 18 years; (2) having mild to moderate autism diagnosed at least once by a medical professional; (3) adequate skills for participation in dog activities (The ability to, with minimal verbal or gestural prompting from the teacher; absence of significant aggressive or disruptive behaviors); (4) the legal guardians and teachers of the children were available to answer the proposed questionnaires. Participants were excluded when they were (1) allergic to animals, afraid of animals, or had an aversion to animals and (2) had severe mental/cognitive issues that might lead to injuries/inconvenience to the animals and an increased likelihood of undesired events. The primary diagnosis of the children was ASD, and they also had comorbidities like intellectual disability, attention deficit hyperactivity disorder, and behavior disorders. The study was approved by the Institutional Review Board of the University of Hong Kong/Hospital Authority Hong Kong West (approval number: UW 19–131). Before study implementation, informed written consent was obtained from the parents of the participants. The Consolidated Standards of Reporting Trials (CONSORT) reporting guideline was followed in reporting this study.

### The instructor-handler-dog therapy team

The experienced special education teacher, acted as the supervisor, in this study has 53 years of experience working with SEN children and has also completed dog–human interaction courses offered by the Hong Kong Guide Dogs Association (HKGDA). The special education teacher is an essential team member in the DAT training process, taking charge of selecting learning objectives, designing student-dog activities, and documenting and assessing the performance of each student. In the most important part, the special education teacher (the supervisor) also participated in the dog-children interaction throughout the training (eight weekly sessions, each lasting approximately 30 min) and gave an individual report for each child after the training.

The dog handler involved is an experienced female dog handler, equipped with all basic entry skills in the DAT after completing at least 10 h of formal continuing education every year in the topics of animal-assisted intervention, dog training/handling, or dog behavior. She demonstrated positive examples of human interaction, using social skills appropriate to the ASD children in the study. Before the program implementation, she was well informed and introduced to the DAT program, to understand her role in each session when working with participants and the special education teacher. The training was provided by the Hong Kong Guide Dogs Association (HKGDA), according to the Standards of Practice for Dog Handlers in AAI. This standard outlines comprehensive requirements for dog handlers in Animal-Assisted Interventions (AAI). Handlers must demonstrate exemplary human interaction skills, including empathy, adaptability, and effective communication with participants and professionals. They require a thorough understanding of canine behavior, learning theory, and positive reinforcement techniques. Key responsibilities include the following: conducting participant pre-screening for allergies, phobias and risk factors; maintaining animal welfare as primary priority through appropriate session planning and stress monitoring; advocating for the dog’s needs and removing them from unsuitable situations; collaborating with therapeutic professionals to align sessions with participant goals. Competency assessment must evaluate their ability to handle dogs effectively in relevant environments; recognize canine stress signals and health concerns; implement infection control protocols; use appropriate equipment and positive training methods. The standards emphasize the handler’s dual responsibility for participant safety and animal welfare, requiring judicious decision-making to maintain therapeutic integrity while protecting all involved parties.

The AAI dogs were from HKGDA and were selected for docile nature, obedience training, and socialization skills. A thorough evaluation was also approved by veterinary surgeons and animal behavior specialists. The chosen dogs were up to date with relevant vaccinations, including *Bordetella*, and had current immunization records and anti-parasitic treatments. A veterinarian also conducted quarterly clinical examinations of the AAI dogs to ensure physical and emotional health. The AAI dog was based in-house at the selected SEN school for 1 week before the program to assess which children showed no fear of the dog.

### Setting and training procedures

The intervention group received eight consecutive training sessions (twice a week, 1 h/week) with a professional DAT team. The control group received regular school courses without the dog. Post-intervention assessments were conducted after the treatment sessions (using the same assessment tool as the baseline assessment). Within the DAT group, the intervention process was videotaped during the therapy session to record their performance. The DAT sessions were conducted in the activity classroom of the SEN school, a familiar environment for the participants. The therapy consisted of eight weekly sessions, each lasting approximately 30 min, and was performed with pairs of students within the DAT group who possessed similar general abilities. The treatment team consisted of an experienced special education teacher, a teacher assistant, the therapy dog, and the dog handler. The AAI dog had access to a blanket as a resting place. The participants and the therapy team sat in a row, in front of the dog, being close and within easy reach of the dog. Also present during the whole training session was a research assistant, responsible for videotaping. The therapy sessions were tailored to the children under review, depending on their baseline ability. The DAT program aimed primarily to facilitate the generalization and application of existing or emerging social-communicative and behavioral skills by embedding their practice within the motivating and novel context of interactions with a therapy dog. The training program targeted three aspects of each child’s performance: language, behavior, as well as human and dog bonding. The language improvement goal is achieved by understanding and following instructions, answering basic questions, learning new vocabulary, and expanding sentences. The behavior scheme aims at increasing participants’ eye contact and attention span, practicing turn-taking, and contacting new objects (e.g., dog, dog-related objects). During these sessions, a close bonding relationship developed with the dog, the students in the same group, and other members of the therapy team.

Supplementary Table provides specific training program steps. Firstly, the students were required to match name cards with the real person represented in the training team. Interactions between the therapy dog and the children were guided by the dog handler and the teacher who provided education to the children on how the therapy dog liked to be touched e.g., “gentle patting on her back” and “soft brushing”. Student-therapy dog interactions included 6 main approaches. Students were guided to touch the AAI dog and comb the dog’s hair to improve their sensation and upper extremity stimulation. Feeding the dog with a plate putting treats in front of the dog, holding the dog leash, and walking with the dog helped students to participate in the activities of daily living and mobility. Children’s engagement in socialization and interesting activities, such as instructing the dog to retrieve a ball, is essential. Navigating the AAI dog through various obstacles enhances agility. To improve their communication ability, students were asked to put labels, stickers, or clips on the dog’s scarf. Next, the teacher gave a demonstration or verbal cues to students about how to give orders to the therapy dog, for example, “OK”, “go”, “go to the bed”, and “hand-hand”. Students then imitated the teacher and took turns to play interactive games. The special education teacher encouraged the student to say the command out loud and gave some verbal or physical prompts when the student indicated. On completing the session, the therapy team encouraged each child to speak up in praise of themselves, to enhance their confidence. The children were also supported by the dog handler, who was an experienced professional in managing the dog throughout each session. At the end of each session, the students and therapy team played pretend photo shoots together, such as looking at the camera, smiling and making sign gestures. All participants then said “bye-bye” to each other to close the therapy activity.

### Questionnaire

#### Pediatric quality of life inventory (PedsQL)

The PedsQL 4.0 Generic Core Scales (parent report for children ages 8–12) was employed to assess parents’ perceptions of their child’s health-related quality of life (HRQOL) [20, 21]. The PedsQL has been extensively employed and validated among pediatric populations, confirming its appropriateness for assessing health-related quality of life in this particular group. It has shown strong reliability, validity, sensitivity, and responsiveness for both child self-reports (ages 5–18) and parent proxy reports (ages 2–18) [22, 23]. The 23 items of the PedsQL are distributed in four dimensions evaluating physical functioning (8 items), emotional functioning (5 items), social functioning (5 items), and school functioning (5 items). Items were rated on a 5-point Likert scale ranging from 0 (never) to 4 (almost always). Scores were transformed to a scale from 0 to 100. Items are reverse scored and linearly transformed to a 0–100 scale as follows: 0 = 100, 1 = 75, 2 = 50, 3 = 25, 4 = 0, so that higher scores corresponded with better HRQOL. The psychosocial health summary score is computed as the sum of the items over the number of items answered in terms of the emotional, social, and school functioning scales. The physical health summary is defined as the overall score of the physical functioning subscale.

#### Strengths and Difficulties Questionnaire (SDQ)

The SDQ (one-sided for teachers of 4–17 years old) is an instrument composed of 5 scales (each with 5 items) and it aims to evaluate children’s mental health in terms of emotional symptoms, conduct problems, hyperactivity, peer problems, and prosocial behavior during the past 6 months [24, 25]. The SDQ has been extensively used in ASD research and has demonstrated acceptable psychometric properties for screening emotional and behavioral problems in this population, though it is recognized as a broader mental health screening tool [26]. The overall difficulties score of the parent and teacher SDQ demonstrates acceptable validity and reliability across all groups, regardless of informant, child’s gender, or parental education level [27]. For each of the 5 scales, the score ranges from 0 to 10 based on three alternatives for ranking the statements: “somewhat true” (1 point), “not true” and “certainly true” varies with the item (0 point or 2 points). Total difficulties score is generated by summing scores from all the scales except the prosocial subscale. The externalizing score is the combined total of the conduct and hyperactivity scales, while the internalizing score comprises the emotional and peer problems scales. A high score represents greater difficulties, except for the prosocial behavior score, for which a lower score indicates greater difficulties.

### Statistical analysis

To ensure data integrity, a double-data entry procedure was employed. Furthermore, all statistical analyses were conducted by a researcher who was blinded to group allocation (DAT vs. control) to prevent potential bias during the analysis. The normality of the distribution for quantitative variables was evaluated using the Kolmogorov–Smirnov test. Variables with a normal distribution were expressed as mean ± standard deviation (SD), while continuous data with skewed distribution were presented as the median (IQR). Gender, age, and baseline questionnaire scores were described in the DAT group and control group and were compared by independent *t*-test or chi-square test. Normality test of raw data of scores, the change difference of pre- and post- scores of PedsQL and SDQ was conducted by Kolmogorov–Smirnov test. For within-group changes: To evaluate the hypothesis that DAT leads to significant improvement over time within the intervention group, paired-samples *t*-tests were used for normally distributed pre-to-post change scores on the PedsQL and SDQ. The normality of these change scores was confirmed using the Kolmogorov–Smirnov test. For between-group comparisons, To test the primary study hypothesis, the post-intervention scores and change difference on the PedsQL and SDQ were compared between the DAT and control groups. Independent samples *t*-tests were employed when the data met assumptions of normality and homogeneity of variance. If these assumptions were violated, the non-parametric Mann–Whitney *U* test was used as a robust alternative. All the analyses were conducted using SPSS version 29.0, with a two-sided significance level of *α* = 0.05.

## Results

Characteristics of participants included in the study are presented in Table 1. The majority of children in this study are male, accounting for 78.1% (25/32) in the DAT group, and 87.5% (28/32) in the control group. The mean age of the DAT group and the control group is 10.3 years and 10.8 years, respectively. There was no significant difference between the two groups in gender and age. Before the intervention, children’s quality of life, described as PedsQL, and psychosocial difficulties, represented as SDQ, had no significant difference between the two groups (all *P* > 0.05) (Table 1).

**Table 1:** Characteristics of participants included in the study at baseline

Characteristics	DAT group (*n* = 32)	Control group (*n* = 32)	*P*
Gender, *n* (%)			0.508
Female	7 (21.9)	4 (12.5)	
Male	25 (78.1)	28 (87.5)	
Age, years, mean (SD)	10.3 (1.98)	10.8 (2.00)	0.382
PedsQL, mean (SD)			
Pre_Physical functioning	66.1 (16.3)	67.4 (22.0)	0.794
Pre_Emotional functioning	64.4 (14.9)	65.5 (19.9)	0.804
Pre_Social functioning	42.2 (24.2)	42.5 (24.6)	0.959
Pre_School functioning	60.6 (17.2)	59.7 (16.0)	0.822
Pre_Psychosocial summary score	57.1 (13.6)	55.9 (16.7)	0.754
Pre_Total Score	58.3 (13.5)	58.8 (15.8)	0.370
SDQ, mean (SD)			
Pre_Total difficulties score	15.6 (5.86)	16.5 (5.88)	0.568
Pre_Externalizing problems score	7.84 (3.85)	8.16 (3.79)	0.745
Pre_Internalizing behavior score	7.78 (2.98)	8.31 (3.25)	0.498
Pre_Emotional problems score	2.84 (2.17)	2.78 (1.91)	0.903
Pre_Conduct problems score	2.25 (1.95)	2.12 (1.60)	0.780
Pre_Hyperactivity score	5.59 (2.70)	6.03 (2.73)	0.521
Pre_Peer problems score	4.94 (1.81)	5.53 (2.53)	0.285
Pre_ Prosocial behavior score	4.12 (2.64)	3.69 (2.67)	0.512

The normality test indicated that the scores for PedsQL and SDQ were normally distributed, while the differences between pre- and post-scores were non-normal distribution. Table 2 shows the results of the pre- and post-mental health scores in the two groups. In the DAT group, sub-scores of physical functioning and school functioning were significantly improved, from 66.11 to 71.09 and 60.63 to 67.50, respectively. This also led to the mean score improvement in the psychosocial summary (pre vs. post: 57.08 vs. 61.25, *P* = 0.043) and the total score (pre vs. post: 58.32 vs. 63.71, *P* = 0.025). The majority of subscale scores of the SDQ assessment also showed significant improvement in the DAT group after intervention. The mean score of total difficulties was significantly reduced from 15.63 to 13.16 after intervention (*P* = 0.003). The mean score of externalizing problems decreased from 7.84(3.85) prior to the intervention to 6.22(3.84) after intervention (*P* = 0.004) attributed to the reduced mean score of conduct problems (pre vs. post: 2.25 vs. 1.47, *P* = 0.010) and hyperactivity (pre vs. post: 5.59 vs. 4.75, *P* = 0.015). In the control group, all the subscale score changes before and after the intervention were not statistically significant (all *P* > 0.05). No statistically significant changes were observed in terms of the SDQ mental health subscale scores except an evident reduction of the total difficulties sub-score, decreasing from 16.47(5.88) to 15.03 (5.03) (*P* = 0.035).

**Table 2:** Comparison of mental health score pre- and post-intervention in the DAT or control group

Group	Variables	Pre		Post		*P*
		Mean	SD	Mean	SD	
DAT group (*n* = 32)						
	PedsQL					
	Physical functioning	66.11	16.35	71.09	19.20	**0.058**
	Emotional functioning	64.38	14.85	68.13	16.30	0.161
	Social functioning	42.19	24.19	48.13	22.42	0.101
	School functioning	60.63	17.17	67.50	14.31	**0.005**
	Psychosocial summary	57.08	13.56	61.25	14.39	**0.043**
	Total score	58.32	13.52	63.71	14.22	**0.007**
	SDQ					
	Total difficulties	15.63	5.86	13.16	6.02	**0.003**
	Externalizing behavior	7.84	3.85	6.22	3.84	**0.004**
	Internalizing behavior	7.78	2.98	6.81	3.68	0.128
	Emotional problems	2.84	2.17	2.25	2.26	0.166
	Conduct problems	2.25	1.95	1.47	1.54	**0.010**
	Hyperactivity	5.59	2.70	4.75	2.77	**0.015**
	Peer problems	4.94	1.81	4.69	2.10	0.517
	Prosocial behavior	4.13	2.64	4.44	2.82	0.370
Control group (*n* = 32)						
	PedsQL					
	Physical functioning	67.38	22.00	69.14	20.23	0.510
	Emotional functioning	65.47	19.93	65.31	18.36	0.964
	Social functioning	42.50	24.59	47.81	25.11	0.286
	School functioning	59.69	15.96	63.59	18.80	0.221
	Psychosocial summary	55.89	16.74	58.91	15.90	0.316
	Total score	58.76	15.77	61.46	15.73	0.350
	SDQ					
	Total difficulties	16.47	5.88	15.03	5.03	**0.035**
	Externalizing behavior	8.16	3.79	7.38	3.10	0.111
	Internalizing behavior	8.31	3.25	7.66	3.32	0.135
	Emotional problems	2.78	1.91	2.34	1.91	0.218
	Conduct problems	2.13	1.60	1.88	1.48	0.317
	Hyperactivity	6.03	2.73	5.50	2.29	0.127
	Peer problems	5.53	2.53	5.31	2.22	0.490
	Prosocial behavior	3.69	2.67	3.63	2.14	0.847

Comparison results of mental health scores between the two groups after intervention are displayed in Table 3. Though the difference between the two groups was not statistically significant (all *P* > 0.05), all the subscale scores or total scores of PedsQL in the DAT group after the intervention were higher than that in the control group and all the post-scores of the SDQ in the DAT group was lower compared with the control group. After the intervention, the mean total score of PedsQL was 63.71(14.22) in the DAT group, which was higher than the score of 61.46(15.73) in the control group. The mean score [13.16(15.03)] of total difficulties of the DAT group was lower than the mean score [15.03 (50.3)] in the control group. Table 4 shows the post–pre difference of mental health scores between the two groups. There were no statistically significant change differences between the two groups (all *P* > 0.05). However, the increased total score of the DAT group [median (IQR): 100(− 87.5,243.75)] was higher than that of the control group [median (IQR): 87.5(− 68.75, 256.25)]. The reduction of total difficulties [median (IQR): − 2(− 4.75,0)] of the AAI group was lower than that of the control group [median (IQR): − 1(− 4, − 0.25)].

**Table 3:** Comparison of mental health scores between the two groups after intervention

Variables	DAT group (*n* = 32)		Control group (*n* = 32)		*P*
	Mean	SD	Mean	SD	
PedsQL					
Post_Physical functioning	71.09	19.20	69.14	20.23	0.693
Post_Emotional functioning	68.13	16.30	65.31	18.36	0.519
Post_Social functioning	48.13	22.42	47.81	25.11	0.958
Post_School functioning	67.50	14.31	63.59	18.80	0.353
Post_Psychosocial summary	61.25	14.39	58.91	15.90	0.539
Post_Total score	63.71	14.22	61.46	15.73	0.783
SDQ					
Post_Total difficulties	13.16	6.02	15.03	5.03	0.181
Post_Externaling behavior	6.22	3.84	7.38	3.10	0.190
Post _Internalising behavior	6.81	3.68	7.66	3.32	0.339
Post_Emotional problems	2.25	2.26	2.34	1.91	0.858
Post_Conduct problems	1.47	1.55	1.88	1.48	0.286
Post_Hyperactivity	4.75	2.77	5.50	2.29	0.242
Post_Peer problems	4.69	2.10	5.31	2.22	0.252
Post_ Prosocial behavior	4.44	2.82	3.63	2.14	0.198

**Table 4:** Comparison of the post–pre difference in mental health score between the two groups (Mann–Whitney *U* test)

Variables	DAT group (*n* = 32)	Control group (*n* = 32)	*P*
Post–Pre, PedsQL, median (IQR)			
Post–Pre Physical functioning	6.25(− 5.47,14.84)	1.56(− 6.25,12.5)	0.531
Post–Pre Emotional functioning	5(− 5,15)	0(− 10, 17.5)	0.454
Post–Pre Social functioning	2.5(− 10,25)	5(− 10, 15)	0.973
Post–Pre School functioning	5(0,10)	10(− 3.75, 15)	0.569
Post–Pre Psychosocial summary	2.50(− 4.58,14.58)	3.33(− 1.25, 11.67)	0.701
Post–Pre Total score	5.94(− 4.14,13.01)	2.81(− 2.46, 12.46)	0.773
Post–Pre, SDQ, median (IQR)			
Post–Pre Total difficulties	− 2(− 4.75,0)	− 1(− 4, − 0.25)	0.445
Post–Pre Externalizing behavior	− 1(− 3,0)	− 1(− 2, 0.75)	0.488
Post–Pre Internalizing behavior	− 1(− 3.75,1)	− 1(− 2, 0.75)	0.787
Post–Pre Emotional problems	− 1(− 2,1)	0(− 2, 1)	0.625
Post–Pre Conduct problems	0(− 1.75,0)	0(− 1, 0)	0.462
Post–Pre Hyperactivity	− 1(− 2,0.75)	− 1(− 2, 0.75)	0.702
Post–Pre Peer problems	0(− 1.75,1)	0(− 1, 1)	0.995
Post–Pre Prosocial behavior	0(− 1,1.75)	0(− 1,1)	0.473

## Discussion

This is the first exploratory RCT in children to explore the effectiveness of DAT on psychosocial well-being and quality of life in children with mild or moderate ASD, employing independent evaluation by both parents and schoolteachers. Our study found that DAT can lead to enhanced psychosocial functioning and quality of life, and the effects were consistent across home and school settings as reported by both parents and teachers. However, DAT displayed a similar interventional effect compared to traditional course training. This preliminary evidence demonstrates the feasibility of such research in a real-life population, showing that the DAT could be a useful complementary/adjunctive therapeutic input for children in receipt of conventional educational training in a SEN school setting. This finding suggests novel ideas and options that can be integrated into a comprehensive treatment and assessment plan for ASD, helping to enrich and improve existing approaches, and the use of more comprehensive intervention approaches, enabling evaluation of the benefit for ASD students from novel inputs in real-life situations.

Our study shows that DAT can significantly improve the quality of life and decrease psychosocial difficulties, particularly behavior problems. Guillen Guzman, E., et al. explored the advantages of utilizing DAT as an additional method in a children’s mental health day hospital. The findings suggested that children who participated in DAT had fewer emotional and behavioral disruptions during their stay at the day hospital, indicating an improved self-regulation ability, self-control, and social response [28]. From the parent’s perspective, they also partly contribute to the progress of ASD children to the improvement of behavioral regulation [3]. In terms of emotion, one study introduced DAT into a pediatric intensive care unit, and findings suggested that it was not only feasible, secure, and widely accepted by both patients and medical staff, but also proved successful in decreasing levels of pain, fear, and anxiety [29]. Relief from negative emotion was also confirmed by another study investigating the stress reduction effect of DAT in SEN children [30]. The development of other skills in the DAT training also indirectly led to the improvement of behavioral and emotional, such as enhanced social participation [10], and improved social communication [13; 19]. Early AAI in preschoolers increases children’s social participation due to the increased child-dog social relationships (look at the dog, touch it, talk to it, and get involved in an activity with the animal) and child-therapist relationships (look at the therapist and talk to him/her) [10].

DAT has a comparable effect to the school’s educational curriculum in improving the psychosocial health and quality of life of children with ASD. When supporting the education of children with ASD, DAT has the potential to serve as a valuable supplementary component alongside traditional educational interventions, contributing to socio-educational development. Currently, limited RCTs were designed to compare the effectiveness of DAT and other conventional therapies on mental health, reporting inconsistent results. Several studies also produced similar effectiveness between the DAT and the conventional mental health interventions. A pilot RCT conducted in Australian children also showed no statistically significant improvement between the groups when compared with the control group, although there was a positive trend for on-task behavior and goal attainment within the dog-involved group [14]. Another study compared physical therapy and dog-involved therapy in clinical recovery in adults, showing improved quality of life, and reduction of pain and anxiety levels in both interventions [31]. Some other studies reported better intervention effectiveness of DAT. One study revealed that the dog intervention greatly enhanced adaptive social and communication abilities in children. This progress was maintained even after the dog training intervention, demonstrating a superior effect compared to the standard-of-care interventions [13]. Another RCT study conducted in adults showed better-reduced stress and symptoms of agoraphobia and improved social awareness and communication in adults with ASD with normal to high intelligence, compared to the conventional intervention [32]. Several potential explanations may contribute to our results. The effectiveness of DAT depends on participants’ compliance with the training program, the frequency and duration of the intervention, the severity of ASD symptoms, and the presence of any comorbidity in children with ASD. In terms of our study, the participation rate for all 8 training sessions was 59.37% (19 out of 32). This was due to 12 students from the DAT group missing between 1 and 3 sessions, with one student only being able to attend one session due to physical health issues. The absence may lead to less effectiveness of DAT on the student represented by the score. Besides, it was difficult for the children to sustain their ability beyond the experimental setting (interaction with the dog) as they appeared directed more toward the special education teacher and the dog rather than their peers. Future studies can focus on developing protocols to encourage cooperation and communication among peers within or beyond the intervention group as well as extend the improvements into other daily life settings.

The focus of the conventional education curriculum and DAT was different, though no statistical difference was found, which targets different aspects of ability when incorporating the DAT for children with ASD. DAT emphasizes emotional and psychological intervention. The mechanism of the DAT on children with ASD mainly works through the interaction between humans and dogs. The companionship of dogs can stimulate positive emotions in ASD students, alleviating their anxiety and stress [33], and capturing the attention of participants [34]. The behavior and reactions of dogs can also serve as feedback, helping ASD students better understand and express their emotions, and promoting the development of social skills [34]. School-based curriculum in the SEN school targets promoting cognitive and language development (enhancing learning abilities, language, and communication skills), improving social skills and emotional management, developing self-care abilities, enhance behavior management. In the implementation process, a special educational curriculum requires professional teachers and teaching facilities, while DAT requires a professional team and facilitators. Therefore, within special educational curriculum, we can also apply the DAT to children with ASD.

The effectiveness of DAT on the psychological well-being of children with ASD may vary across different difficulty subscales. Our result shows that DAT seems more effective in reducing behavior problems than in reducing emotional problems or enhancing prosocial behavior. Our study finds a significant decrease of the subscale score for externalizing behaviors (conduct problems and hyperactivity) in our DAT group. This research reinforces findings from earlier observations using the parent-rated Child Behaviour Checklist (CBCL), where children with ASD displayed a reduction of internalizing problems, externalizing problems, and the total problem scale after the therapy sessions [35]. While the presence of a therapy dog can provide temporary relief and promote relaxation [28], it may not be sufficient to address the underlying causes of internalizing behaviors. These behaviors may require more targeted interventions that delve deeper into the individual’s thoughts, emotions, and experiences. Our study also suggests that the impact of DAT on enhancing prosocial behavior may be limited. This finding is consistent with another study conducted on psychiatrically hospitalized youth with ASD [19]. It may be that the intervention was context-specific and did not address the items on the assessment of the SDQ questionnaire (e.g., able to empathize with others’ feelings, happy to share things with other kids, etc.). These aspects were not built into the DAT program and may be out of context. This negative outcome and discrepancy can also be attributed to the small sample size, which may prevent the conduction of statistical analyses with accurate conclusions. One other possibility is that there is a delayed effect of the DAT on prosocial behavior, and the reduction of ASD symptoms might not immediately result in improved prosocial skills, as suggested by a previous study [36]. Long-term tracking of participants’ prosocial behavior changes is necessary to further understand this aspect. It is possible that our dog-involved program, with 30-min sessions once a week, for a total of 8 sessions, may not be intensive enough to effectively improve prosocial behavior in the longer term. A recent review by Nieforth et al. [7] reported a striking variability in animal-assisted intervention (AAI) programs, with the number of sessions ranging from 1 to 80, session length from 20 min to 3 h, and total contact time from 3 to 100 h. This review primarily focused on studies involving equine, canine, and dolphin interventions, but also included other animals in the analysis. The minimum number of AAI sessions required to observe improvements in behavior or skills remains unknown [37]. Previous studies suggest treatment may not be effective in ameliorating poor prosocial skills, implying the ‘intractability’ of autism [38]. The effect of AAI on prosocial behavior (more helpful and empathetic) has primarily been reported in healthy adolescents, increasing prosocial behavior rather than in individuals with ASD [39].

One of the strengths of our study is its randomized designed with a representative sample across Hong Kong as the sample source covers its three administrative regions. Another notable strength was the collection of independent assessments of mental health by both schoolteacher and parents who were blind to each other’s assessments.

It is important to acknowledge several limitations in this study. Firstly, the sample size might have been too small to detect a significant difference between the two groups. A larger sample size could have provided more statistical power to identify even small but meaningful effects of the interventions. Secondly, the short follow-up time of the study made assessing possible long-term effects impossible. Thirdly, the study did not fully consider individual differences, such as the severity of the condition, subject age, and gender, previous and concurrent exposure to dogs or other pets and their impact. Fourthly, bias of changes in outcomes measured by parents and schoolteachers, it would be better to evaluate the severity of core ASD symptoms with standard scales. Fifthly, there is no standardized training consensus for AAI or DAT to date. This study employed an empirical approach designed by an experienced SEN teacher. It would be beneficial to refine the structure of the DAT intervention and develop a protocol to assess these designs in the Hong Kong setting and then use it to promote the pilot study to a more generalized population. Lastly, there could be other differences between the DAT group and comparison group that were not identified. Other potential moderators, such as individual ability, severity of ASD, and other comorbidities should also be considered when analyzing the effectiveness of DAT on the improvement of mental health of children with ASD if detailed data information was collected.

Future research can expand the sample size and include ASD children of different ages and severity levels, to improve the universality and reliability of the research results. At the same time, the follow-up time of the study can be extended to observe the long-term effects of the two intervention methods. In addition, further exploration of the impact of individual differences on intervention effects can be conducted, providing a basis for the design of personalized treatment plans. For example, for ASD individuals of different ages, learning objectives and dog-assisted therapy programs suitable for their developmental stages to improve the treatment effect. Further studies are needed to explore the long-term effects and how to sustain this effect beyond the therapy setting. Besides, the positive effect of simply participating in a research study is a crucial methodological consideration that could contribute to the similarity in outcomes between groups. Future research would benefit from a mixed-methods approach, incorporating qualitative interviews or focus groups with participants and their families. This would provide a richer, more nuanced understanding of the therapeutic process, the perceived benefits, and the potential influence of the research context itself, thereby helping to interpret quantitative outcomes. Besides, incorporating a questionnaire or standardized tool to evaluate barriers to participation and social satisfaction in children would be valuable for identifying individual or contextual factors that may affect the success of DAT in future studies. While employing RCTs enhances the internal validity of our results concerning DAT’s effectiveness, it is important to recognize that the interpretation of these findings is limited by the specific outcome measures selected. Relying primarily on parent-reported questionnaires, which are useful for capturing perceived changes in quality of life and psychosocial functioning, may not fully reflect more subtle behavioral or physiological shifts. Future research should expand on this RCT approach by adopting a multi-method assessment strategy that includes direct observations, physiological measurements, and evaluations of potential moderating factors to gain a more detailed understanding of how and for whom DAT is most beneficial.

## Conclusions

Our preliminary finding shows the DAT has a similar effect on improving the quality of life and reducing social difficulties compared to the conventional curriculum in children with ASD. That means the DAT, as a complementary intervention option, can be incorporated into conventional training courses. Future studies should focus on larger-scale, long-term randomized controlled experiments involving a more extensive sample from various settings. This approach would promote the generalizability of the results, offer a more robust evidence base for the intervention’s effectiveness, and ascertain its sustained impact on improvements throughout and beyond the therapy session.

## Supplementary Information

Below is the link to the electronic supplementary material.

ESM1 (DOCX 16.0 KB)

## Data Availability

No datasets were generated or analysed during the current study.
