# Correction to: Dog-assisted therapy on Hong Kong children with autism spectrum disorder: an exploratory randomized controlled trial

**DOI:** 10.1007/s00431-026-06762-4

**Published:** 2026-01-29

**Authors:** Wilfred H. S. Wong, Chen Chen, Amy Tso, Hung Kwan So, Justin P. Y. Wong, Helen Tinsley, Charis H. Y. Chung, Ronda K. W. Luk, Patrick Ip

**Affiliations:** Department of Paediatrics and Adolescent Medicine, School of Clinical Medicine, Li Ka Shing Faculty of Medicine, University of Hong Kong, Room 115, 1/F, New Clinical Building, Queen Mary Hospital, 102 Pokfulam Road, Hong Kong, SAR China

**Correction to: European Journal of Pediatrics (2026) 185:64**

10.1007/s00431-025-06720-6

In the article “Dog-assisted therapy on Hong Kong children with autism spectrum disorder: an exploratory randomized controlled trial” published in the *European Journal of Pediatrics*, an error appears in the “What is New” section of the abstract.

The following sentence was incorrectly included:

“Most cases in pediatric outbreaks occurred among healthcare workers pointing to the need to protect HCW from infections and a limited role of pediatric patients and caregivers.”

This sentence is unrelated to the study and was not provided by the authors.

It should be replaced with the following correct statement:

“DAT may serve as a valuable complementary therapy for children with ASD alongside conventional curriculum-based training.”

The original article has been corrected.

