# Supplementary material for: Dog-assisted therapy on Hong Kong children with autism spectrum disorder: an exploratory randomized controlled trial
Source: Eur J Pediatr. 2026 Jan 9;185(1):64. doi: 10.1007/s00431-025-06720-6 (PMC12789236; doi:10.1007/s00431-025-06720-6)
Supplement: Supplementary file 1 — (DOCX 16.0 KB) [file 431_2025_6720_MOESM1_ESM.docx]

| **Supplementary Table. Learning objectives and the activities of the dog-assisted therapy programme.** | |
| --- | --- |
| **Learning Objectives** |  |
| Language | 1. Understand and follow instructions  2. Answer Questions, Who? What? What does he/she/it do? How? Why? What next?  3. Learn new vocabulary  4. Expand sentence |
| Behaviour | 1. Increase eye contact  2. Increase attention span  3. Practise turn-taking  4. Contact new objects (dog, dog-related objects)  5. Follow instructions  6. Interact with classmates |
| Human and dog bonding | 1. Build a relationship with new friends  2. How to interact with the dog  3. Develop close contact with the dog |
| **The activities of the Programme** | |
| Sensation and upper extremity stimulation | Touch the AAI dog; comb the AAI dog’s hair |
| Activities of daily living and gait | Feed the AAI dog; walking with the AAI dog |
| Socialisation and entertainment | Playing with the AAI dog, drawing for the AAI dog |
| Agility course | Guide the AAI dog through obstacles |
| Expression course | Put labels, stickers or clips on the AAI dog’s scarf |
| Speech lessons | Give the dog instructions, call the AAI dog’s name |
